# Primary Esophageal Motility Disorders: Beyond Achalasia

**DOI:** 10.3390/ijms18071399

**Published:** 2017-06-30

**Authors:** Francisco Schlottmann, Marco G. Patti

**Affiliations:** 1 Department of Surgery and Center for Esophageal Diseases and Swallowing, University of North Carolina, Chapel Hill, NC 27599, USA; 2 Department of Medicine and Center for Esophageal Diseases and Swallowing, University of North Carolina, Chapel Hill, NC 27599, USA; marco_patti@med.unc.edu

**Keywords:** esophageal motility disorders, high-resolution manometry, Chicago classification

## Abstract

The best-defined primary esophageal motor disorder is achalasia. However, symptoms such as dysphagia, regurgitation and chest pain can be caused by other esophageal motility disorders. The Chicago classification introduced new manometric parameters and better defined esophageal motility disorders. Motility disorders beyond achalasia with the current classification are: esophagogastric junction outflow obstruction, major disorders of peristalsis (distal esophageal spasm, hypercontractile esophagus, absent contractility) and minor disorders of peristalsis (ineffective esophageal motility, fragmented peristalsis). The aim of this study was to review the current diagnosis and management of esophageal motility disorders other than achalasia.

## 1. Introduction

The best-defined primary esophageal motor disorder is achalasia. However, symptoms such as dysphagia, regurgitation and chest pain can be caused by other esophageal motility disorders. Unfortunately, while there is a reasonable consensus on the pathophysiology, diagnosis and treatment of achalasia, this has not occurred for other motility disorders [1].

Esophageal manometry, the best method to evaluate esophageal motility, evolved from intraluminal balloons to water perfused catheters, and then to solid state probes; from unidirectional measurements to circumferential data gathering; and from simple pressure curves to intuitive colorful temporal-pressure plots. This technological evolution led to the development of high-resolution manometry (HRM) [2].

The more comprehensive HRM plots provoked esophageal physiologists to develop new manometric parameters and reclassify esophageal motility disorders. This led to a compilation of significant clinical findings under the Chicago classification [3]. This classification was recently revised (version 3.0) to exclude some previous parameters without clear clinical application, characterize new parameters, and redefine motility disorders [4]. Motility disorders beyond achalasia now include: esophagogastric junction outflow obstruction, major disorders of peristalsis (distal esophageal spasm, hypercontractile esophagus, absent contractility) and minor disorders of peristalsis (ineffective esophageal motility, fragmented peristalsis).

The aim of this study was to review the current diagnosis and management of esophageal motility disorders beyond achalasia.

## 2. HRM Parameters

### 2.1. Contractile Vigor—Distal Contractile Integral

HRM allows the evaluation of contractility through a combination of amplitude, time and length of the peristaltic wave. This parameter is the distal contractile integral (DCI). DCI value is calculated as the product of the mean amplitude of contraction in the distal esophagus (mmHg), the duration of contraction (seconds), and the length of the distal esophageal segment (cm) exceeding 20 mmHg for the region spanning from the transition zone to the proximal aspect of the lower esophageal sphincter (LES). DCI classifies waves as failed (DCI < 100 mmHg/s/cm), weak (DCI 100–450 mmHg/s/cm), ineffective (failed or weak), normal (DCI 450–8000 mmHg/s/cm) or hypercontractile (DCI > 8000 mmHg/s/cm).

### 2.2. Peristalsis—Distal Latency

HRM evaluates peristalsis by the distal latency (DL) which measures objectively the timeframe of the wave from the beginning of the swallow (upper esophageal sphincter relaxation) to an inflection of the peristaltic axis known as the contractile deceleration point. Premature contractions are defined with a DL < 4.5 s.

Fragmented contractions are considered segmental defects (break in the 20-mmHg isobaric contour > 5 cm) with normal contraction vigor.

### 2.3. Lower Esophageal Sphincter Pressure

The Chicago Classification did not define parameters for lower esophageal sphincter (LES) length or basal pressure, but simply recommended assessment of pressure as an average of inspiratory and expiratory values for three normal respiratory cycles. Relaxation, however, is measured not by the nadir pressure, as previously done with conventional manometry, but with the integrated relaxation pressure (IRP), which corresponds to the mean pressure of 4 s of greatest post deglutive relaxation in a 10 s gap, triggered at the beginning of a swallow.

## 3. Esophageal Motility Disorders beyond Achalasia

### Esophagogastric Junction Outflow Obstruction

Esophagogastric junction (EGJ) outflow obstruction is characterized by an impaired LES relaxation (IRP > 15 mmHg) with normal or weak peristalsis.

## 4. Major Disorders of Peristalsis

### 4.1. Distal Esophageal Spasm

**Figure 1:**
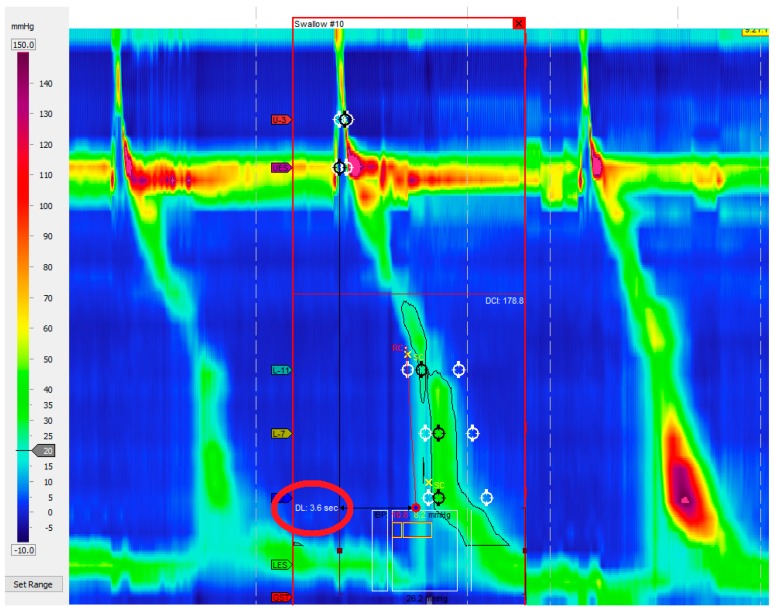
Distal esophageal spasm. Premature contractions (DL < 4.5 s) in at least 20% of swallows.

Distal esophageal spasm (DES) is defined by premature contractions (DL < 4.5 s) in at least 20% of swallows with a normal IRP (Figure 1).

### 4.2. Hypercontractile Esophagus

**Figure 2:**
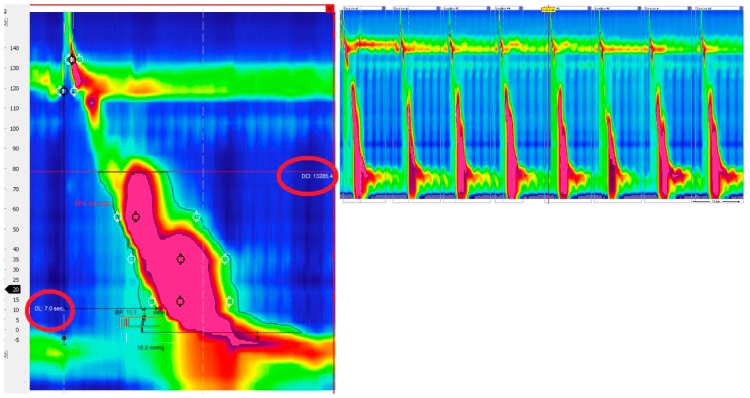
Hypercontractile esophagus (jackhammer esophagus). DCI > 8000 mmHg/s/cm in at least 20% of swallows and normal DL.

Hypercontractile esophagus (jackhammer esophagus) is characterized by DCI > 8000 mmHg/s/cm in at least 20% of swallows and normal DL (Figure 2).

### 4.3. Absent Contractility

**Figure 3:**
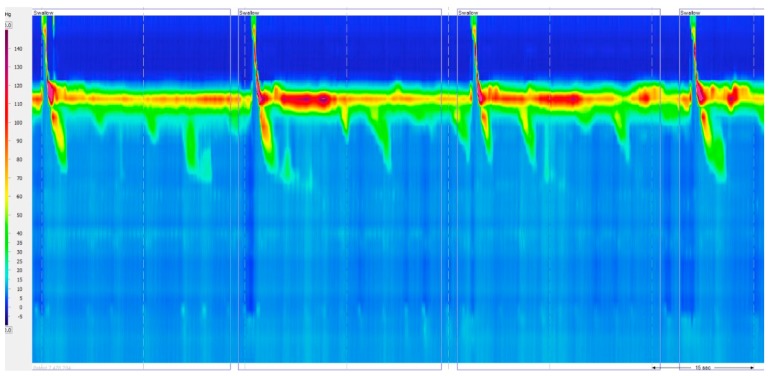
Absent contractility. Aperistalsis in the setting of normal LES relaxation (IRP < 10 mmHg).

Absent contractility is characterized by aperistalsis in the setting of normal LES relaxation (IRP < 10 mmHg) (Figure 3).

## 5. Minor Disorders of Peristalsis

### 5.1. Ineffective Esophageal Motility

**Figure 4:**
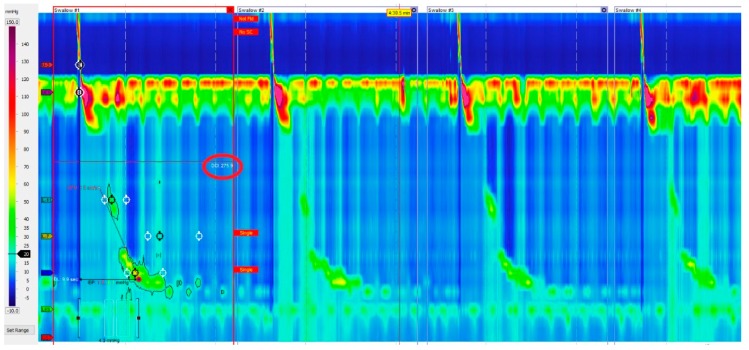
Ineffective esophageal motility. Failed or weak peristalsis in at least 30% of swallows.

Ineffective esophageal motility is defined by ≥ 50% ineffective swallows (failed or weak—DCI < 450 mmHg/s/cm) (Figure 4).

### 5.2. Fragmented Peristalsis

**Figure 5:**
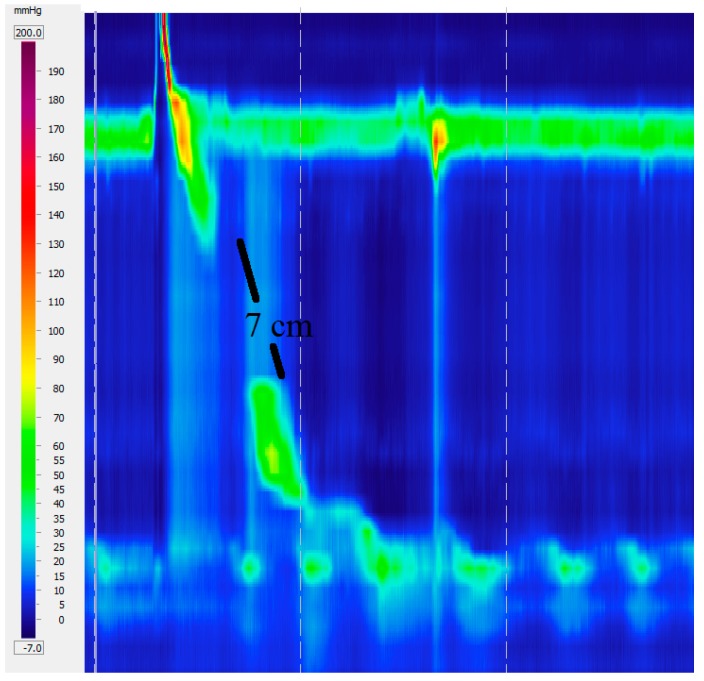
Fragmented peristalsis with a 7 cm gap.

Fragmented peristalsis is defined by ≥50% fragmented contractions with normal contraction vigor (Figure 5).

## 6. Management of Motility Disorders beyond Achalasia

### Esophagogastric Junction Outflow Obstruction

The definition of EGJ outflow obstruction based solely on the IRP with the exclusion of achalasia allows this diagnosis to be superimposed to other diagnosis dependent on the esophageal body motility. It may be caused by an anatomical abnormality at the cardia (hiatal hernia, diseases of the esophageal wall, etc.) or be idiopathic with normal anatomy.

Similar to achalasia, treatment is directed towards relief of the obstruction and can be accomplished by botulinum toxin injection, pneumatic dilatation (PD), laparoscopic Heller myotomy (LHM) or peroral endoscopic myotomy (POEM). Both botulinum injection and PD showed good relief of dysphagia but with ephemeral duration [5]. Scherer and colleagues [6], diagnosed 16 patients (1.6%) with EGJ outflow obstruction in doing 1000 HRM and treated them with botulinum toxin injection, PD or LHM. Only the three patients treated with LHM responded well. Interestingly, Pérez-Fernández et al. [7] reported that over one-third of the patients with EGJ outflow obstruction presented a spontaneous resolution of the symptoms, concluding that surgical treatment should be considered with special caution in these patients. In the setting of an anatomic abnormality such as a hiatal hernia, surgical correction is associated with long-lasting and excellent results [8].

EGJ outflow obstruction is now recognized as a distinct entity in the Chicago Classification. However, the real clinical significance of this diagnosis is still uncertain. In fact, it could be an early or incomplete expression of a variant of achalasia. Thus, the exact significance and clinical management of these patients remains unclear.

## 7. Major Disorders of Peristalsis

As the symptoms and the manometric picture of esophageal motility disorders can be due to gastroesophageal reflux disease (GERD), it is of paramount importance to rule out abnormal reflux by pH monitoring. If GERD is present, either medical or surgical treatment should be directed towards the control of the reflux [9].

The management of DES remains elusive. The Chicago Classification defines this disorder with a new parameter: the distal latency (DL). Previous reports showed good results in patients with “diffuse esophageal spasm” (Richter classification) [10] who underwent LHM with an extended myotomy [11,12]. However, scarce data are available after the new classification defined DES. A review of the publications after this definition became publicized showed that botulinum toxin injection in the esophageal body was superior to placebo to relieve dysphagia in patients with DES, and that POEM is a promising treatment for these patients [13].

The definition of hypercontractile esophagus (jackhammer) was updated in the last version of the Chicago Classification to include only cases with ≥20% of swallows with a DCI > 8000 mmHg/s/cm, excluding a single altered swallow from the definition. Pharmacological relaxation of the smooth muscle with phosphodiesterase-5 inhibitors or anticholinergic agents has shown symptomatic improvement [14]. Studies focusing on surgical therapy for hypercontractile esophagus based on the new classification are not available. Previous reports not using HRM or the Chicago Classification; however, showed acceptable outcomes after surgical myotomy [15,16].

Absent contractility is mostly diagnosed in patients with connective tissue diseases. There is no specific treatment to restore or improve peristalsis in these patients. Associated GERD is usually the target of therapy [17].

## 8. Minor Disorders of Peristalsis

Therapeutic options for ineffective esophageal motility are still limited, as no effective treatment is available to restore impaired esophageal smooth muscle contractility [18]. Treatment directed towards gastroesophageal reflux disease is helpful when dysmotility is secondary to this disease.

The concept of fragmented peristalsis changed radically from previous versions to the version 3.0. Only large breaks (>5 cm) with normal peristalsis are included. This is more clinically relevant, since incomplete bolus transit is observed in 100% of the cases of large breaks but only in 16% of small breaks [19]. It is unclear how to treat this finding since there are no studies focusing on the treatment for this disease under these criteria. There are no studies evaluating changes in the motility pattern after therapy for gastroesophageal reflux disease as well, since both conditions are frequently associated.

## 9. Conclusions

HRM and the Chicago classification certainly contributed to a better definition of esophageal motility disorders beyond achalasia. Although they have a much lower prevalence than achalasia, they need to be considered in the differential diagnosis of a patient presenting with achalasia symptoms. Unfortunately, the real clinical significance and correct management of these motility disorders are still under investigation.
